# Comprehensive and quantitative profiling of lipid species in human milk, cow milk and a phospholipid-enriched milk formula by GC and MS/MS^ALL^

**DOI:** 10.1002/ejlt.201400575

**Published:** 2015-02-24

**Authors:** Elena Sokol, Trond Ulven, Nils J Færgeman, Christer S Ejsing

**Affiliations:** 1 Department of Biochemistry and Molecular Biology, VILLUM Center for Bioanalytical Sciences, University of Southern DenmarkOdense, Denmark; 2 Department of Department of Physics Chemistry and Pharmacy, University of Southern DenmarkOdense, Denmark

**Keywords:** High resolution mass spectrometry, Milk, Milk products, Shotgun lipidomics

## Abstract

Here we present a workflow for in-depth analysis of milk lipids that combines gas chromatography (GC) for fatty acid (FA) profiling and a shotgun lipidomics routine termed MS/MS^ALL^ for structural characterization of molecular lipid species. To evaluate the performance of the workflow we performed a comparative lipid analysis of human milk, cow milk, and Lacprodan® PL-20, a phospholipid-enriched milk protein concentrate for infant formula. The GC analysis showed that human milk and Lacprodan have a similar FA profile with higher levels of unsaturated FAs as compared to cow milk. In-depth lipidomic analysis by MS/MS^ALL^ revealed that each type of milk sample comprised distinct composition of molecular lipid species. Lipid class composition showed that the human and cow milk contain a higher proportion of triacylglycerols (TAGs) as compared to Lacprodan. Notably, the MS/MS^ALL^ analysis demonstrated that the similar FA profile of human milk and Lacprodan determined by GC analysis is attributed to the composition of individual TAG species in human milk and glycerophospholipid species in Lacprodan. Moreover, the analysis of TAG molecules in Lacprodan and cow milk showed a high proportion of short-chain FAs that could not be monitored by GC analysis. The results presented here show that complementary GC and MS/MS^ALL^ analysis is a powerful approach for characterization of molecular lipid species in milk and milk products.

**Practical applications:**

: Milk lipid analysis is routinely performed using gas chromatography. This method reports the total fatty acid composition of all milk lipids, but provides no structural or quantitative information about individual lipid molecules in milk or milk products. Here we present a workflow that integrates gas chromatography for fatty acid profiling and a shotgun lipidomics routine termed MS/MS^ALL^ for structural analysis and quantification of molecular lipid species. We demonstrate the efficacy of this complementary workflow by a comparative analysis of molecular lipid species in human milk, cow milk, and a milk-based supplement used for infant formula.

## Introduction

Milk and milk products are important sources of dietary lipids during childhood. Milk lipids serve both as nutrients for energy metabolism and as structural building blocks for tissue growth and development. Lipids can be divided into several lipid categories and classes based on their chemical structures [1]. The most abundant lipid class in milk is triacylglycerol (TAG) followed by a lower proportion of phospholipids [2]. These lipids are made up of a multitude of individual molecular lipid species having different fatty acid (FA) moieties linked to a common glycerol-backbone. The molecular composition of lipid species in milk differs among animals [3,4]. Human milk lipids contain a lower proportion of saturated and short-chain FAs and more polyunsaturated FAs as compared to cow milk. The importance of dietary polyunsaturated FAs during pregnancy, lactation, or childhood is highlighted by their beneficial effects on childhood neurodevelopment [5]. For example, improvement in cognitive function has been linked to supplementation of infant formula with glycerophospholipids containing polyunsaturated FA moieties [6]. In addition, supplementation with the phospholipids sphingomyelin (SM) and phosphatidylcholine (PC) have also been reported to protect against gastrointestinal infections and diarrhea during early childhood [7,8]. Notably, the potential health benefits of the molecular composition of individual lipid species in milk and functional milk products remain largely unexplored [9]. A better understanding of the health-promoting and disease-preventive effects of milk lipid composition at the molecular level warrants analytical workflows supporting structural characterization and quantification of molecular lipid species.

Milk lipid analysis is typically performed by FA profiling using gas chromatography (GC) [4,10]. This approach is high throughput-oriented and allows separation and quantification of isomeric FAs having differences in the position and the *cis*/*trans* configuration of double bonds [11]. The methodology requires chemical transesterification whereby both non-esterified FAs and FA moieties of intact lipids are converted to volatile methyl esters (FAMEs). As such, FA profiling by GC analysis fails to track the FA composite of intact molecular lipid species. Moreover, accurate quantification of short-chain FAs is hampered by their higher volatility and extensive losses due to evaporation during sample preparation [12]. Notably, the overall FA composition of distinct lipid classes can be determined using fractionation by solid phase extraction, liquid chromatography, or thin layer chromatography prior to chemical derivatization [13–15]. However, these more cumbersome and time-consuming approaches also do not unequivocally determine the FA composite of individual lipid molecules.

Mass spectrometry (MS)-based shotgun lipidomics is an alternative strategy to quantify individual molecular lipid species and determine their FA composition [16,17]. Shotgun lipidomics implies that lipid extracts are directly infused into a mass spectrometer without up-front time-consuming chromatographic separation, and that identification of lipid species relies on accurately determined masses and/or detection of structure-specific fragment ions. This technique is high throughput-oriented [18,19] and offers a wide dynamic quantification range of up to four orders of magnitude with high analytical sensitivity and specificity [20,21]. A single shotgun experiment allows a reliable quantification of a few hundred lipid species encompassing several lipid classes [20,22–24]. Identified lipid species are annotated by a shorthand nomenclature corresponding to the level of detail attainable by the analysis [25]. As such, detection of lipid species by high resolution MS or lipid class-specific fragment ions (e.g., *m*/*z* 184.0733 for PC species) supports annotation by “sum composition” which denotes the total number of carbon atoms (carbon index) and double bonds (double bond index) in the FA composite (e.g., PC 34:1). In comparison, lipid annotation by more detailed “molecular species composition” (e.g., PC 16:0–18:1, which denotes constituting FA moieties) or by “defined molecular species composition” (e.g., PC 18:1/16:0, which denotes *sn*-1 and *sn*-2 position of FA moieties) requires dedicated tandem mass analysis routines and detection of molecular structure-specific fragment ions [26].

Here we describe a workflow for quantitative and structural characterization of molecular lipid species in milk and milk products. The workflow combines GC analysis for total FA profiling and a shotgun lipidomics routine termed MS/MS^ALL^ for quantitative mapping of molecular lipid species [24]. We demonstrate the efficacy of the workflow through an in-depth and quantitative lipid analysis of human milk, cow milk, and Lacprodan® PL-20, a phospholipid-enriched milk protein concentrate for infant formula. FA profiling by GC analysis showed that human milk and Lacprodan have a similar FA composition featuring higher proportions of unsaturated FAs as compared to cow milk. In contrast, the in-depth characterization by MS/MS^ALL^ revealed that the higher proportion of unsaturated FAs is primarily attributed the composite of TAG species in human milk and glycerophospholipid species in Lacprodan. These results demonstrate that the combined GC and MS/MS^ALL^ workflow is a powerful strategy for in-depth quantitative characterization of the molecular lipid composition of milk and milk products.

## Materials and methods

### Standards and chemicals

FAMEs standard mixture (47885-U) was purchased from Sigma–Aldrich (Copenhagen, Denmark). Lipid standards were obtained from Avanti Polar Lipids (Alabaster, AL). Ammonium acetate and ammonium formate were purchased from Fluka Analytical (Buchs St. Gallen, Switzerland). HPLC grade chloroform and methanol were obtained from Rathburn (Walkerburn, Scotland). *n*-Hexane was obtained from Fisher Scientific (Slangerup, Denmark).

### Annotation of lipid species

Lipid species were annotated either by sum composition or by molecular species composition as previously described [20,27]. Lipid species annotated by sum composition are reported as [lipid class] [total number of carbon atoms in FA moieties]:[total number of double bonds in FA moieties] (e.g., TAG 36:0). Lipid species annotated by molecular species composition are reported as [lipid class] [number of carbon atoms in FA1]:[number of double bonds in FA1]-[number of carbon atoms in FA2]:[number of double bonds in FA2]-[number of carbon atoms in FA3 (only used for TAG)]:[number of double bonds in FA3 (only used for TAG)] (e.g., PC 16:0–18:1, TAG 4:0–16:0–16:0). We note that the symbol “-” denotes only the FA moieties of lipid species, and not their *sn*-1, *sn*-2, and *sn*-3 position on the glycerol-backbone [27,28].

### Milk samples

Human milk was obtained from two volunteers 4 wk post partum. The volunteers were healthy and non-smokers. Cow milk (1.5% fat) and Lacprodan® PL-20 were obtained from Arla Foods (Viby J, Denmark).

### Lipid extraction

For GC analysis, human milk was diluted 3.3 times, cow milk was diluted 5 times, and Lacprodan was dissolved and diluted to a concentration of 8.8 mg/mL in 155 mM ammonium acetate buffer. The samples were extracted at 4°C with 4 mL chloroform/methanol (2:1, v/v) for 60 min under vigorous shaking. The lower organic phase was collected, vacuum evaporated, and the lipid extracts were subjected to FAME conversion (see below). For MS/MS^ALL^ analysis, human milk was diluted 200 times, cow milk was diluted 100 times, and Lacprodan was diluted to the concentration of 0.3 mg/mL in 155 mM ammonium acetate buffer. Total of 200 μL of each sample was added 10 μL of internal standard mixture providing a spike of 93 pmol TAG 17:1–17:1–17:1, 94 pmol phosphatidylethanolamine (PE) 17:0–14:1, 94 pmol phosphatidylserine (PS) 17:0–14:1, 96 pmol PC 17:0–14:1, 98 pmol phosphatidylinositol (PI) 17:0–14:1, and 100 pmol sphingomyelin (SM) 18:1;2/17:0. Subsequently, samples were added 1 mL chloroform/methanol (2:1, v/v) and extracted at 4°C for 60 min under vigorous shaking. The lower organic phase was collected and evaporated, and the lipid extracts were subjected to MS/MS^ALL^ analysis (see below).

### FA profiling by GC analysis

FAMEs were prepared by acid-catalyzed transesterification [12]. GC analysis was carried out using a Clarus 500 Gas Chromatograph (Perkin Elmer, USA) equipped with a flame-ionization detector and a capillary column (TR-FRAME, 60 m × 0.25 mm i.d., 0.25 m film thickness). Helium was used as a carrier gas at a constant flow rate of 0.8 mL/min. Five microliters of sample were injected into the injector with 10:1 split ratio. The column temperature was maintained at 140°C for 5 min and then raised at rate of 3°C per minute up to 240°C and maintained for 20 min. The injection port and detector temperature was set to 250 and 260°C, respectively. Total chromatographic run time was 58 min. Chromatograms were processed using Total Chrome Navigator software and the amounts of identified FAMEs were quantified using calibration curves of known amounts of standards. Statistical analysis was performed by one-way ANOVA with Fisher's least significant difference test using OriginPro 8.5 (OriginLab Corporation, MA).

### Lipid analysis by MS/MS^ALL^

Lipid extracts were dissolved in chloroform/methanol/2-propanol (1:2:4 v/v/v) containing 7.5 mM ammonium formate. Lipid extracted were analyzed by MS/MS^ALL^ [24] using a TripleTOF 5600 (AB SCIEX, Concord, ON) mass spectrometer equipped with a TriVersa NanoMate (Advion Biosciences, Ithaca). The Triversa Nanomate ion source was operated in positive and negative ion mode with a voltage of ±0.96 kV and back pressure of 1.25 psi. MS/MS^ALL^ analysis was programmed to perform sequential MS/MS experiments in 1 amu steps across the precursor *m*/*z* range of 400–1000 and recording time-of-flight (TOF) MS/MS spectra with an *m*/*z* range of 150–1000. Collision energy was set to 45 eV in positive and negative ion mode. Lipid species detected by MS/MS^ALL^ were identified and quantified using LipidView 1.3 beta software (AB SCIEX, Concord, ON) as previously described [24,27]. Statistical analysis was performed as described above.

## Results and discussion

### FA profiling by GC analysis

**Figure 1:**
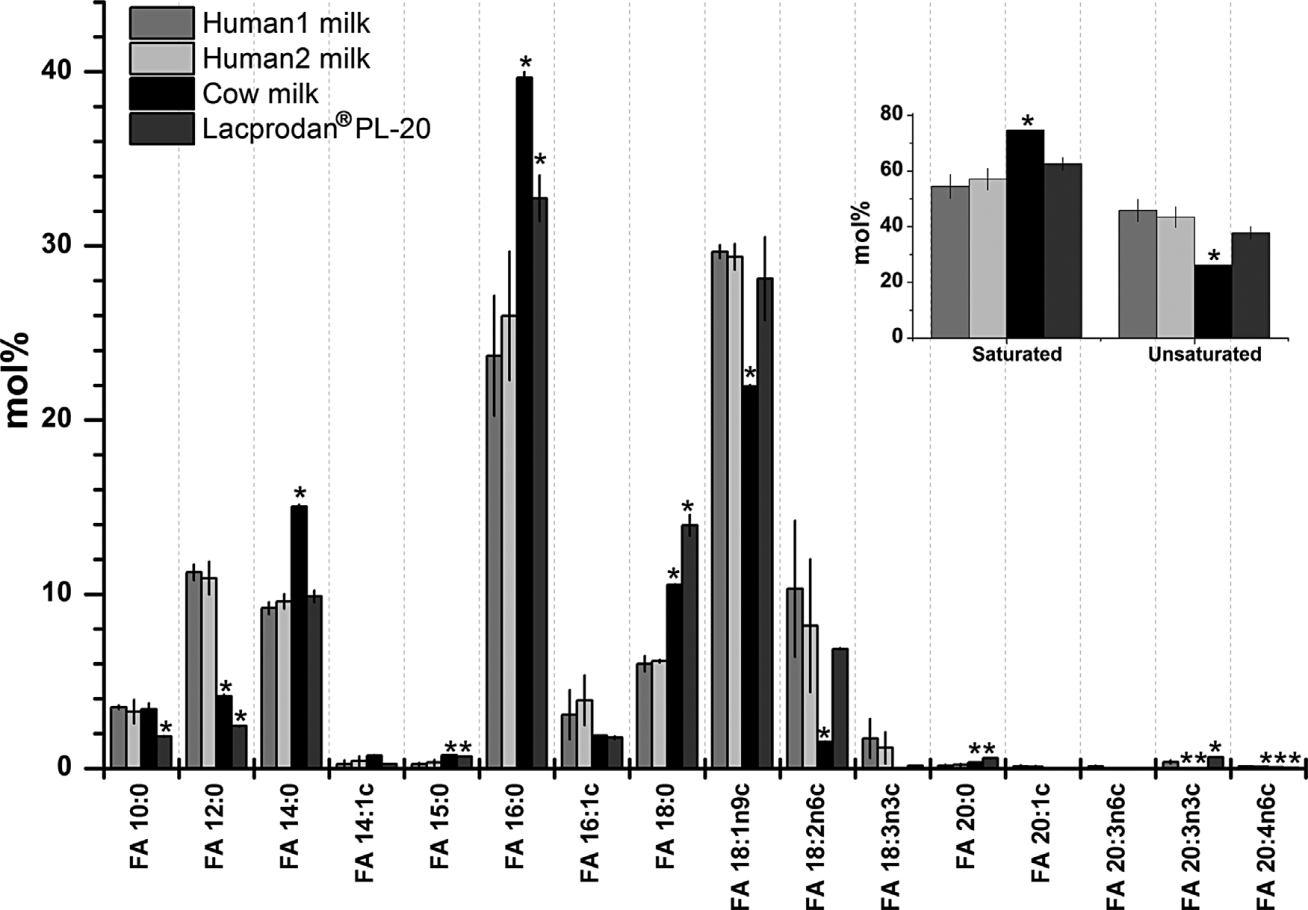
FA profiles of human milk from two volunteers, cow milk, and Lacprodan. Insert: Comparison of levels of saturated and unsaturated FAs. Values represent average ±SD (*n* = 3 replicates). Asterisks (*) denote samples with FA abundances that are significantly different (p-value ≤0.01) as compared to the sample “Human1 milk.” Pairwise comparisons for all samples are reported in Supplementary Table S1.

We first determined the FA profile of human milk, cow milk, and Lacprodan. By GC analysis we identified and quantified 16 FAs having from 10 to 20 carbon atoms (Fig. 1). In each of the three types of milk samples, we observed that the most abundant species were FA 16:0 and FA 18:1n-9c, constituting 55–60% of all quantified FAs. We also observed that human milk and Lacprodan were similar in the overall proportion of unsaturated FAs whereas cow milk contained a significantly higher proportion of saturated FAs (Fig. 1). In particular, human milk and Lacprodan contained more FA 18:2n6c than cow milk. Moreover, we found that each type of milk sample featured a specific pattern of FAs. Human milk contained significantly more FA 12:0, FA 18:3n3c, and FA 20:3n3c as compared to cow milk and Lacprodan. In comparison, cow milk contained more FA 14:0, FA 16:0, and FA 18:0 as compared to human milk. Notably, Lacprodan contained more FA 18:0 than both human milk and cow milk. We note that short-chain FAs with less than ten carbons could not be monitored by the GC analysis. In summary, the GC analysis showed that human milk and Lacprodan were similar in terms of FA composition and overall level of unsaturated FAs.

### Lipid analysis by MS/MS^ALL^

**Figure 2:**
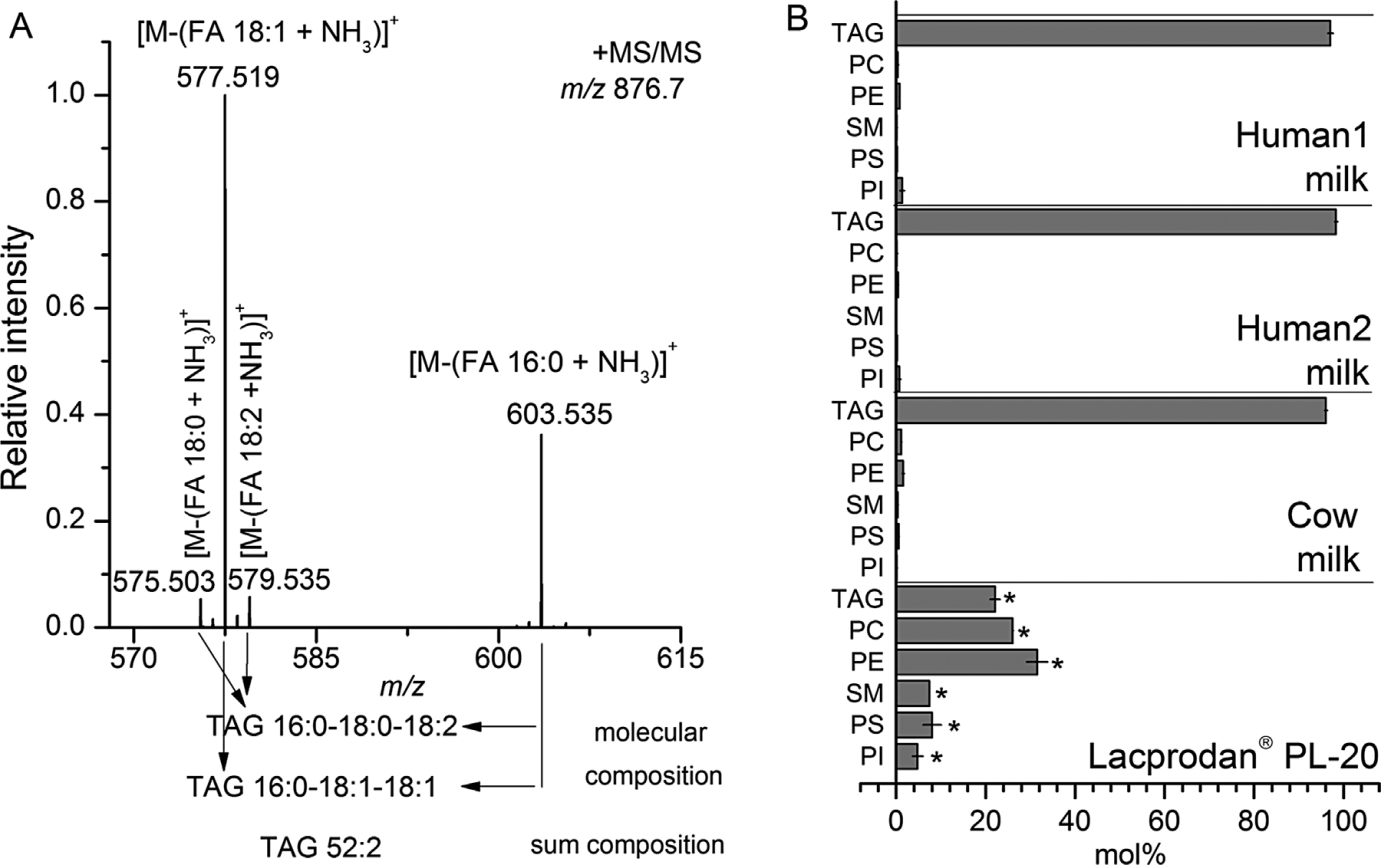
(A) Positive ion mode TOF MS/MS spectrum of *m*/*z* 876.7, corresponding to ammoniated TAG 52:2. Fragment ions at *m*/*z* 575.503, *m*/*z* 577.519, *m*/*z* 579.535, and *m*/*z* 603.535 are correspond to the neutral loss of FA 18:0, FA 18:1, FA 18:0, and FA 16:0, respectively. (B) Lipid class composition of human milk, cow milk, and Lacprodan. Values represent average ±SD (*n* = 3 replicates). Asterisks denote lipid classes with abundances that are significantly different (p-value ≤0.01) as compared to the sample “Human1 milk.” Pairwise comparisons for all samples are reported in Supplementary Table S2.

**Figure 3:**
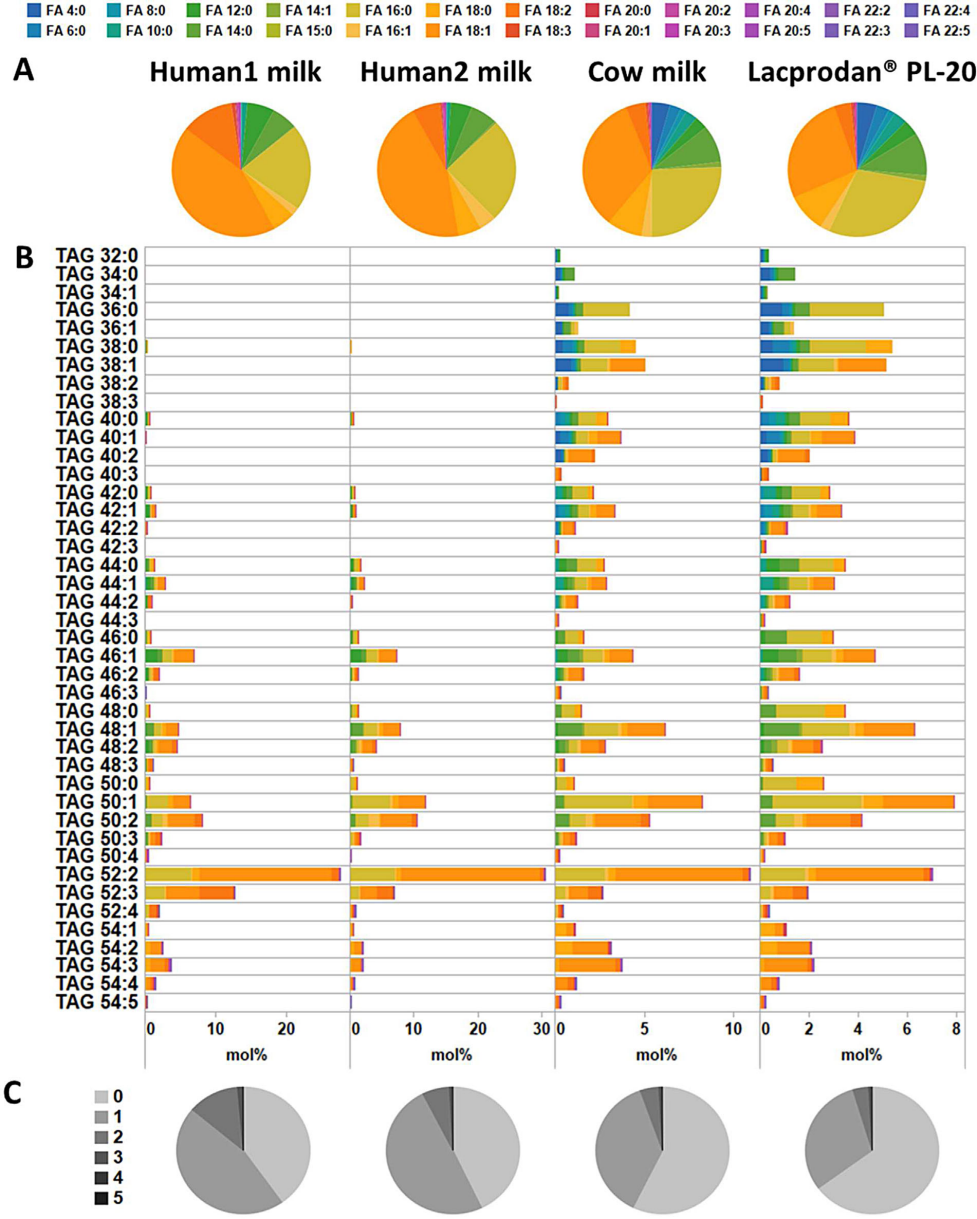
Composites of TAG species in human milk, cow milk, and Lacprodan. (A) FA profile of TAG species. Data represent the percentage of individual FAs in all TAG molecules. (B) Composition of TAG species. Color coding shows the FA index for each TAG species (i.e., the proportion of each FA esterified to a TAG species annotated by sum composition). (C) FA unsaturation index for TAG species. Color coding shows the proportion of FAs with 0–5 double bonds. All values represent an average of three replicate analyses.

Next we examined the composition of molecular lipid species in the three types of milk samples. To this end, we performed shotgun lipidomic analysis using MS/MS^ALL^ on a hybrid quadrupole TOF mass spectrometer [24]. The MS/MS^ALL^ analysis allows unbiased acquisition of high resolution TOF MS/MS spectra for all potential lipid species across a selected mass range. Subsequent processing of spectral data by dedicated software allows identification and quantification of molecular lipid species [27,29]. For example, TOF MS/MS of ammoniated TAG species produces structure-specific fragment ions corresponding to the neutral loss of FA moieties, which in turn enables identification of their hydrocarbon chain length and number of double bonds [29–31]. Fragmentation of *m*/*z* 876.7, corresponding to ammoniated TAG 52:2, yielded fragment ions at *m*/*z* 575.503, 577.519, 579.535, and 603.535 matching the neutral loss of FA 18:0, FA 18:1, FA 18:2, and FA 16:0, respectively (Fig. 2A). This pattern of fragment ions shows that the TAG 52:2 is a composite of the isomeric and molecular species TAG 16:0–18:1–18:1 and TAG 16:0–18:0–18:2. The MS/MS^ALL^ approach, as well as other shotgun lipidomic methods, supports accurate quantification of TAG species annotated by sum composition. This is done by calculating the sum of fragment ion intensities corresponding to the neutral loss of attached FA moieties and normalizing it to the intensity and spike amount of the internal TAG standard (e.g., the sum of fragment ion intensity from neutral loss of FA 18:0, FA 18:1, FA 18:2, and FA 16:0 is used to quantify the absolute amount of TAG 52:2) [27]. In comparison, the quantification of isomeric molecular TAG species by shotgun lipidomics requires deconvolution strategies that aims to resolve intensities of common fragment ions (e.g., neutral loss of FA 16:0 from both TAG 16:0–18:1–18:1 and TAG 16:0–18:0–18:2) and concomitantly estimate the stoichiometry between isomeric TAG species [29,32]. Such strategies use assumptions about the fragmentation behaviors of molecular TAG species that can be useful for estimating the abundance of molecular TAG species provided only a few isomeric molecules are present. For sample matrices with more complex compositions of molecular TAG species, such as milk, the accuracy of such strategies become questionable as the assumptions about TAG fragmentation behavior are not valid due to dependencies on the hydrocarbon chain length, degree of unsaturation and the position on the glycerol backbone of the FA moieties [33–35]. An alternative approach to quantify FA heterogeneity in isomeric and molecular TAG species is to compute a FA index. This index is based on the abundances of fragment ions matching the neutral loss of FA moieties derived from distinct TAG species with a given sum composition (see below, Fig. 3B). We note that quantification of molecular glycerophospholipid species with two FA moieties is relatively straightforward as compared to molecular TAG species [27]. Moreover, we also note that the MS/MS^ALL^ analysis does not provide information about the exact position of FA moieties on the glycerol-backbone nor the positions of double bonds in the FA moieties.

Comparative analysis of human milk, cow milk, and Lacprodan by MS/MS^ALL^ allowed the identification of 136 molecular lipid species encompassing 6 lipid classes (i.e., TAG, PC, PE, PS, PI, and SM). Comparison of molar abundance showed that TAG is the most abundant lipid class in human milk and in cow milk, constituting more than 97 mol% of all quantified lipids (Fig. 2B). Lacprodan contained a lower molar abundance of TAG species (25 mol%) which is comparable to the levels of PE (35 mol%) and PC (18 mol%) species in this sample. For Lacprodan we also detected SM, PS, and PI species (8, 9, and 5 mol%, respectively). In summary, the MS/MS^ALL^ analysis demonstrated that human milk and cow milk are comparable on the lipid class level whereas Lacprodan is, as indicated by the manufacturer, enriched in phospholipids.

### Composition of TAG species

The MS/MS^ALL^ analysis identified and quantified 42 distinct TAG species (annotated by sum composition) having FA moieties with 4 to 20 carbon atoms and having from 0 to 5 double bonds. Importantly, the MS/MS^ALL^ analysis afforded the detection of fragment ions corresponding to the neutral loss of short-chain FA moieties (i.e., FA with 4 to 10 carbon atoms), which could not be monitored by GC analysis (Fig. 3A,B). Manual inspection of TOF MS/MS data showed a high heterogeneity of FA moieties for majority of TAG species annotated by sum composition. For each TAG species we found on average 5–6 distinct fragment ions corresponding to the neutral loss of a FA moiety. As such, each TAG species with a given sum composition is composed of approximately 3–4 isomeric molecular TAG species. To assess the FA profile across all TAG species we used the MS/MS^ALL^ data to estimate the percentage of individual FA moieties irrespective of their originating TAG species (Fig. 3A). This analysis showed that the abundance of short-chain FAs was considerably lower in human milk (∽1.5% of all TAG molecules) as compared to that of cow milk and Lacprodan (∽12–13%). These short-chain FAs were primarily composites of TAG molecules with a low carbon index: e.g., TAG 36:0, TAG 38:0, and TAG 38:1 (Fig. 3B). In contrast, TAG species in human milk were mostly composed of molecules with a higher carbon index: e.g., TAG 52:2, TAG 52:3, and TAG 50:2, which predominately contained FA 16:0 and FA 18:1 moieties as indicated by their FA index (Fig. 3B). Assessing the overall FA unsaturation index of TAG molecules across the three types of milk samples demonstrated that human milk contained a higher proportion of mono-unsaturated (∽45–50%) and di-unsaturated FA moieties (∽6–12%) in comparison to both cow milk and Lacprodan (Fig. 3C). In contrast, cow milk and Lacprodan contained a higher proportion of TAG species with saturated FA moieties (∽57–65%). We note that the evaluation of unsaturation index for only TAG molecules by GC analysis would require fractionation of TAG class lipids prior to the chemical derivatization. Taken together, the MS/MS^ALL^ analysis revealed that TAG molecules in human milk were composed of more unsaturated and longer chain FAs as compared to both cow milk and Lacprodan, which were comprised of a higher proportion of TAG molecules with saturated and short-chain FA moieties.

### Molecular composition of glycerophospholipid species in Lacprodan

**Figure 4:**
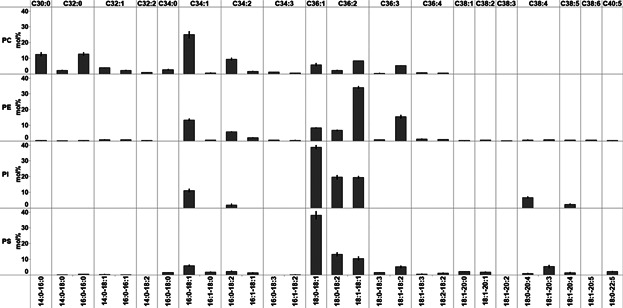
Molecular composition of glycerophospholipid species in Lacprodan. Values represent average ±SD (*n* = 3).

Next we evaluated the molecular FA composite of glycerophospholipids in Lacprodan. By MS/MS^ALL^ we identified and quantified 77 molecular glycerophospholipid species (Fig. 4). The composition of molecular glycerophospholipid species featured primarily FA 16:0, FA 18:0, FA 18:1, and FA 18:2 moieties. As such, the most abundant species were PC 16:0–18:1 (23 mol%), PE 18:1–18:1 (34 mol%), PI 18:0–18:1 (39 mol%), and PS 18:0–18:1 (38 mol%). Glycerophospholipid species with polyunsaturated FA moieties, e.g., FA 20:4 (arachidonic acid) and FA 22:5 (docosapentaenoic acid), were primarily identified as PE, PS, and PI species with molar abundance of less than 5 mol% across all monitored lipid classes. In comparison to TAGs, glycerophospholipid species contained higher proportion of longer and more unsaturated FAs (Supplementary Figure S1). No glycerophospholipid species with short-chain FA moieties (less than 12 carbon atoms) could be detected.

## Conclusions

Here we presented a workflow for in-depth analysis of milk lipids. The workflow uses a combination of GC analysis for FA profiling and shotgun lipidomics using MS/MS^ALL^ analysis for quantification and structural characterization of molecular lipid species. Integrating these approaches into a single workflow provides complementary datasets that report a multitude of compositional lipid features ranging from overall FA levels to quantification of individual molecular lipid species. We demonstrated the efficacy of the workflow by reporting on the lipid composition of human milk, cow milk and Lacprodan® PL-20, a phospholipid-enriched milk concentrate used for infant formula. The results showed that the three types of milk samples featured a unique lipid composition both when assayed using GC analysis and by the MS/MS^ALL^ approach. Notably, human milk comprised a high proportion of TAG molecules with unsaturated and longer-chain FAs. In contrast, cow milk featured a heterogeneous pool of TAG molecules with both saturated short-chain FAs, and unsaturated longer-chain FAs. Interestingly, the supplement Lacprodan comprised a FA profile resembling that of human milk when assayed by GC analysis. However, in-depth MS/MS^ALL^ analysis showed that the similarity on the total FA level was attributed the composite of glycerophospholipids in Lacprodan and TAG molecules in human milk. Notably, the use of MS/MS^ALL^ revealed both compositional and structural features of milk lipids that GC analysis alone cannot ascertain. We conclude that the workflow developed here is a generic approach for milk lipid analysis that can be used for complementing functional studies of the health promoting effects of milk lipids and serve as a quality control routine.

## Abbreviations

FA: fatty acid
FAME: fatty acid methyl ester
GC: gas chromatography
MS: mass spectrometry
PC: phosphatidylcholine
PE: phosphatidylethanolamine
PI: phosphatidylinositol
PS: phosphatidylserine
SM: sphingomyelin
TAG: triacylglycerol

## Notes

Supporting information available online http://dx.doi.org/10.1002/ejlt.201400575
