# Supplementary material for: Comprehensive and quantitative profiling of lipid species in human milk, cow milk and a phospholipid-enriched milk formula by GC and MS/MSALL
Source: Eur J Lipid Sci Technol. 2015 Feb 24;117(6):751–9. doi: 10.1002/ejlt.201400575 (PMC4463771; doi:10.1002/ejlt.201400575)

**Supporting Information for**

Comprehensive lipid profiling of human milk, cow milk and a phospholipid-enriched milk protein concentrate for infant formula

Elena Sokol^1^, Trond Ulven^2^, Nils J. Færgeman^1^ and Christer S. Ejsing^1^

^1^Department of Biochemistry and Molecular Biology, University of Southern Denmark, Odense, Denmark.

^2^Department of Department of Physics, Chemistry and Pharmacy, University of Southern Denmark; Odense; Denmark.

**Table S1.** Pairwise comparisons of mean FA abundances monitored by GC analysis. Significant differences determined by one-way ANOVA with Fisher's least significant difference test (p-value≤0.01) are indicated with “S” in the column “Significance”. SEM, standard error of the mean.

**Table S1** (continued)

**Table S1** (continued)

**Table S2**. Pairwise comparisons of mean TAG, PC, PE, PS, PI and SM class abundances monitored by MS/MS^ALL^. Significant differences determined by one-way ANOVA with Fisher's least significant difference test (p-value≤0.01) are indicated with “S” in the column “Significance”. SEM, standard error of the mean.

**Figure S1.** Comparison of FA profiles of all lipids, all TAG species and all glycerophospholipid species in Lacprodan. Data for all lipids was obtained by GC analysis. Data for all TAG species and all glycerophospholipid species was obtained by MS/MS^ALL^ analysis. Note that no FAs with hydrocarbon chain length less than 10 were monitored by GC analysis. Color coding shows the proportion of each FA.


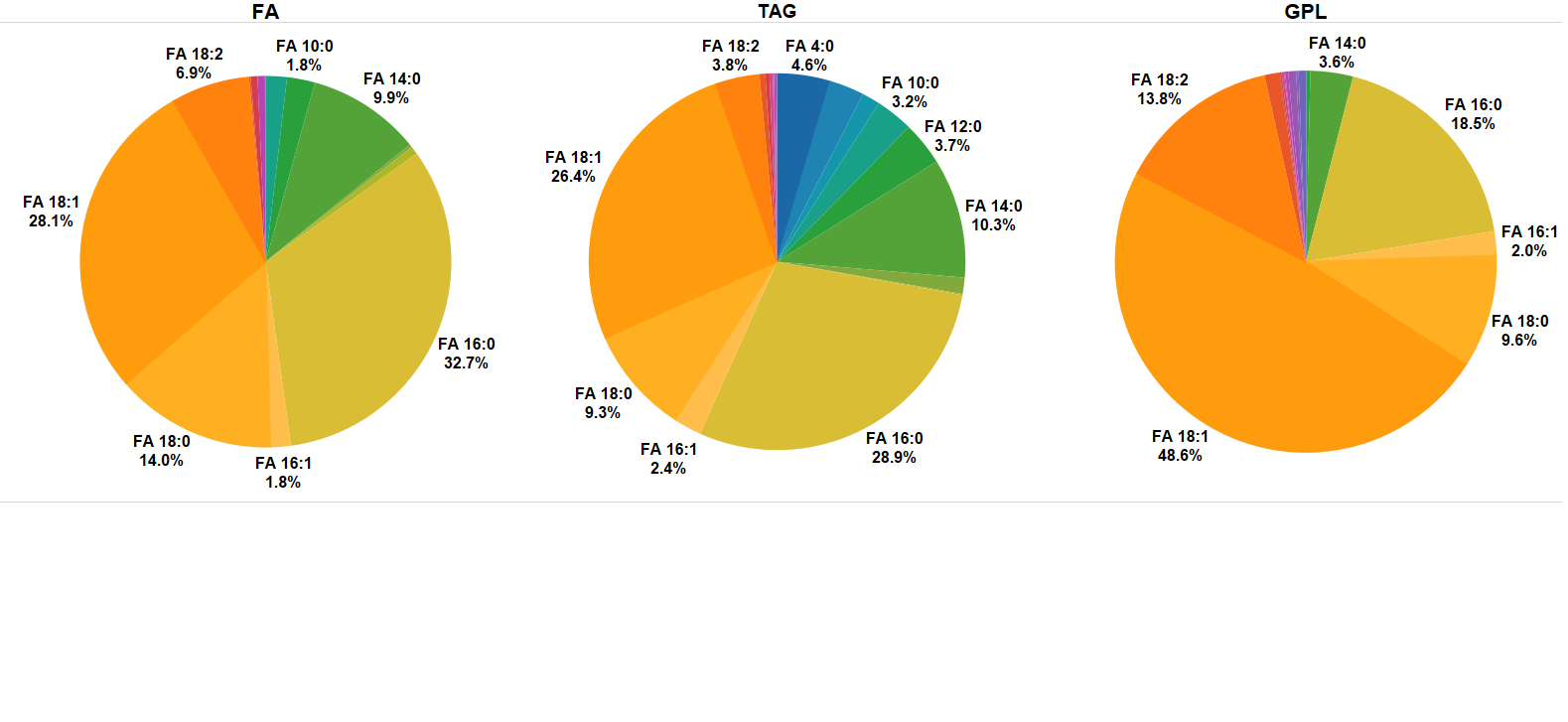

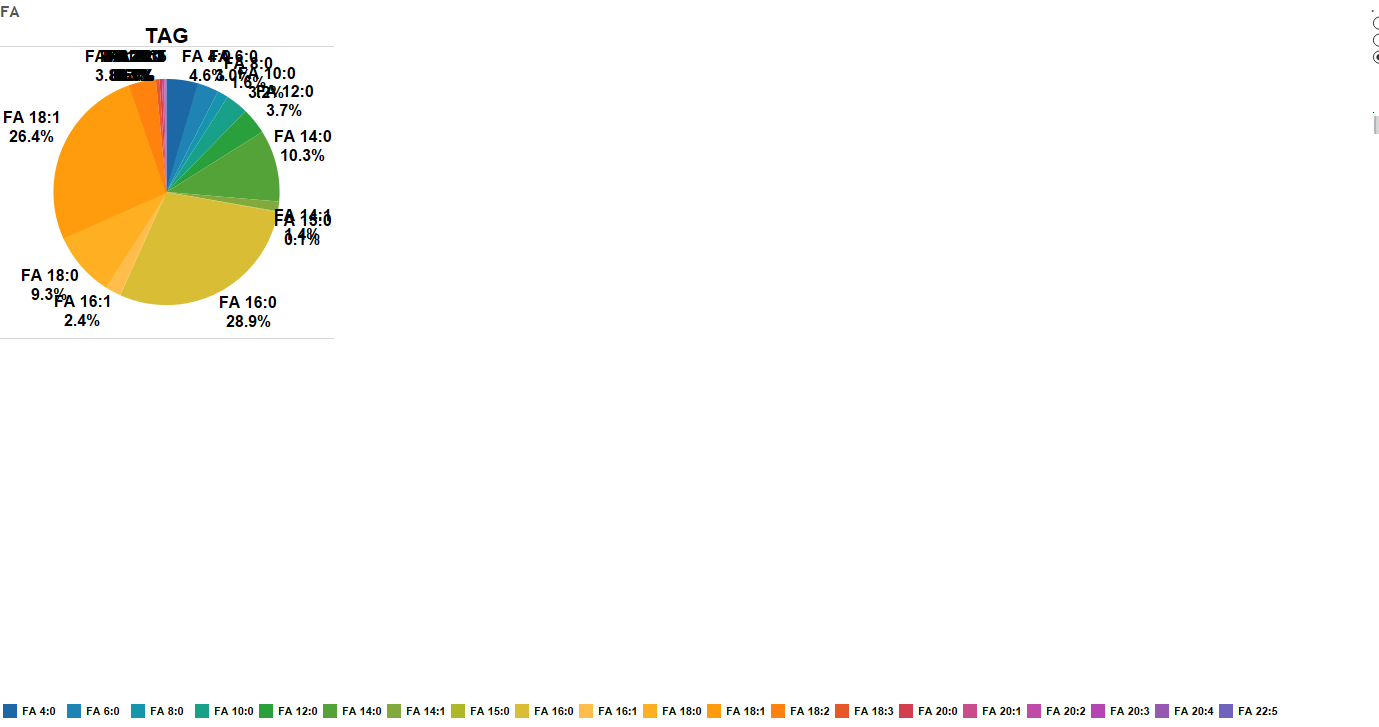

Supplement: Supplementary file 1 [file ejlt0117-0751-sd1.docx]
